# Prediction Model and Data Simulation of Sports Performance Based on the Artificial Intelligence Algorithm

**DOI:** 10.1155/2022/7238789

**Published:** 2022-10-07

**Authors:** Guang Lu

**Affiliations:** School of Sports and Physical Education, Shandong Sport University, Rizhao 276826, Shandong, China

## Abstract

There is still a certain deviation between the current artificial intelligence technology and the traditional learning mode, which makes it unable to be effectively applied in teaching and learning. Therefore, an effective method needs to be proposed to use functions to predict data. Function calculation can not only solve the complex problems of data calculation process but also make the data evenly distributed to take full advantage of the capabilities of each system. In this experiment, we mainly use the control function. After substituting the data into the control function, the function will automatically classify the data. In this paper, according to the actual situation of physical education in colleges and universities, from the two aspects of artificial intelligence and comprehensive learning algorithm, to build a system which can collect and analyze the past achievements of college students' physical education performance simulation can effectively help the design of physical education curriculum. According to the distribution of experimental data, a specific conclusion can be drawn; that is, the test model we choose can calculate and measure the physical fitness level of students, but there are big differences. In contrast, our experimental method using the ensemble computing model can not only predict and analyze the physical fitness level of college students but also reduce errors and shorten the time required for the experiment.

## 1. Introduction

The integrated-learning model refers to combining multiple systems so that they can serve the learning mechanism together. By combining the advantages of many learning systems, a more optimized learning method can be obtained so that the model can promote the development of students [1]. In terms of analyzing data, the new model is significantly better than the previous model. This report is based on the integration model and proposes a relatively novel classification method in the ADA system. The specific experimental steps are as follows: The first step is to extract samples. We first select *n* items related to the integrated system in the database. Then substitute the data into the FAD system to start the functions in each system [2]. The second step uses the classification mechanism in different systems to divide the selected data into several categories and save them in the hardware. This can avoid the information interference that is encountered during transmission, resulting in data loss, but in this way, the calculation steps of the computer will increase, and the calculation difficulty of the system will increase. Finally, we substitute the data divided into several categories into the ADA system to get more detailed results after the division [3]. Through specific experiments, we can know that the integrated model has many advantages and can improve the shortcomings of the previous model so that the student's sports performance data can be more accurate and more practical suggestions can be obtained. People's material level is getting higher and higher, which makes people's demand for the quality of life also increasing, but with the emergence of smart devices such as mobile phones, people often spend a lot of time on entertainment ignoring the development of their own physical health. Nowadays, few people take physical exercises. We focus on the physical activities of college students. Through the collection and analysis of college students' physical education courses, which enable students to enjoy physical activities, we understand that physical test results are also an important resource for studying the development of students' physical fitness [4].

To make the experiment successful, you need to focus on the problem of aggregation. The aggregation mode is different from the data collection and classification mode [5]. It integrates the data with the same characteristics, and then the characteristics of the data are significantly different. This process is automated and does not require the supervision and assistance of any equipment. The final classification result is composed of many connected sets. When we analyze the collection data, because each category does not have its own fixed name, we need to code according to the characteristics of the collection and then arrange them in order. When encoding, we can use the MAC system, which is the fastest and most concise algorithm [6]. This report focuses on the application of artificial intelligence in integrated algorithm systems. The physical performance of college students will be different due to different ages, genders, and physical conditions. They are unstable, but the types of differences are more stable and typical. Therefore, we combine theory with neuroscience to design a collection of students, which is a new way of testing performance in sports. The first step is to design a new model that is generally suitable for the analysis of college students' sports performance, then fix the initial data and the maximum calculated value in the model to minimize the error, and finally analyze the experimental results based on the calculated data.

## 2. Related Works

Literature believes that we should focus on the design of the new model [7]. Only when the model is designed accurately can the final sports test results be accurate. Therefore, researchers have put forward many questions about the completeness and accuracy of the experiment. There are many ways to answer the questions. One of the methods is a diversified experimental model, but because the model is greatly affected by function calculations, the data output is relatively unstable, so it is impossible to accurately predict sports performance. Literature proposes a method for predicting the physical fitness of students through a neural network scientific system [8]. This method is not affected by the function model and can be well combined with linear systems. However, the system is only partially effective in the category but not strong in analyzing the overall results. Literature proposes to predict students' sports performance through a linear network system, which can improve the predictive ability of some functions, but linear network systems are often affected by signals and have high environmental requirements, so there are also shortcomings [9]. The conclusion of the literature is that when we use this model for data analysis room, we need to combine these functions and then perform data calculations, so as to avoid the problems of traditional models and make the effect of processing data more obvious [10]. Literature believes that if we only analyze from the overall point of view, data deviation is easy to occur, so the data classification obtained has problems, and the system can easily delete positive data as irrelevant data if the test result is wrong [11]. Literature found that today's model establishment can already deal with the above-mentioned problems, and the performance of the system is relatively good, mainly to solve the problems of inaccurate data classification and unbalanced time distribution [12]. However, for more complex situations, it is necessary to change the overall experimental method. While updating the system, the selection of hardware equipment must also be changed to a certain extent. This method should be the most optimized method at present. Literature compares the traditional experimental model with the newly designed experimental model and improves the shortcomings of the traditional experimental model so that the data can be distributed in various systems in a more balanced manner [13]. The optimized new experimental model has a high use value and experimental value. Literature believes that in order to make the experiment more complete, it is necessary to divide the entire system into different parts first and then perform a final analysis according to the classification results so that not only can the functions of each system be fully utilized but also deleting duplicate data has made us a major improvement in the balanced distribution of data [14].

## 3. Integrate the Learning Algorithm and Artificial Intelligence Algorithm Prediction Model Establishment

### 3.1. Integrated Learning Algorithm

Nowadays, people have introduced artificial intelligence technology in many fields, which allows people to use new methods to learn and make learning more profound. This is also an important area of research by many scholars. Function calculations can not only solve the complex problems of data calculation process but also make data evenly distributed and make full use of the functions of each system. In this experiment, we mainly use the control function. After substituting the data into the control function, the function will automatically classify the data. We can know that if the result of the data output is closer to the result we initially predicted, it means that the experimental model is relatively successful. After getting improved, it can be applied to specific experiments, and the system consumes less energy. However, if the data we get is far from the data predicted at the beginning, it means that some functions of this mode have problems. It is difficult for us to classify and analyze the input data through this mode. Therefore, we cannot use this mode for experiments. When we process image information, we can know that different images have different resolutions and sizes. If we do not process the original image and convert the image into data, the system will recognize that there is a great difficulty because the memory occupied by the image is much larger than the number, so the system can only calculate from the overall angle when identifying, and there is no way to analyze it in detail, and some images will still be unrecognizable. The loss function mainly solves the situation that the amount of data is too large and the data are more complex, so the loss function has more advantages than the control function in calculation, and the loss function can also solve the problem of the image occupying too much memory, making the data classification more detailed At the same time, we can also add a new loss function as an aid, which can speed up the experiment. The specific function formula is as follows:

(1) $$\begin{array}{c} CE\left(p_{i}\right)=-\alpha_{t}\log\left(p_{i}\right)\text{.} \end{array}$$

Although the method of cross calculation can temporarily calculate the general results of the data, it ignores many small but very critical issues. In the calculation process, the cross calculation is easy to be confused by the data with decimal points, and data errors occur. There is no way to introduce this kind of calculation method with large error into the experiment. Therefore, in order to make the cross calculation method more accurate, we upgrade the system to make the function calculation dominant. The cross calculation is in the function formula based on the data analysis, and we also introduce new factors so that the two complement each other and can better solve the computational problems. The specific principles are as follows:

(2) $$\begin{array}{c} FL\left(p_{i}\right)=-{\left(1-p_{i}\right)}^{\gamma }\;\log\left(p_{i}\right)\text{,}\\ FL\left(p_{i}\right)=-\alpha {\left(1-p_{i}\right)}^{\gamma }\;\log\left(p_{i}\right)\text{.} \end{array}$$

In the process of function calculation, the greater the influence of the initial data on the cross calculation, the greater the error index. On the contrary, the smaller the influence of the initial data on the cross calculation, the smaller the error index, which means the higher the accuracy.

In order to make the data classification of the experiment more reasonable, we conducted data sample experiments on different types of data sets. We analyzed the characteristics of the data with less data in the set and then added the data to those with similar characteristics according to the characteristics. In the collection, this can reduce the amount of data calculation and the type of analysis. At the same time, we have to upgrade the system so that the new system can adapt to the new classification set, then draw conclusions through calculations, and then compare the latest conclusions with the most original data. From the results, we can know that when the classification group decreases, the accuracy of sample analysis increases, and the difficulties encountered in the experiment process are relatively reduced. The latest sample analysis formula is as follows:

(3) $$\begin{array}{c} D_{i+1}\left(x\right)=\frac{D_{i}\left(x\right)e^{-\alpha_{i}f\left(x\right)h_{i}\left(z\right)}}{Z_{i}}. \end{array}$$

We combine the function calculation and the updated system to get a simpler calculation method as follows:

(4) $$\begin{array}{c} D_{i+1}\left(x\right)=\frac{D_{i}\left(x\right)e^{-\alpha_{i}f\left(x\right)h_{i}\left(z\right)f{\left(1-p_{i}\right)}^{2}}}{Z_{i}}. \end{array}$$

The next step is to design a specific experimental step. We selected a number of experimental sets as the initial data and import these data into the UCI system for calculation. The purpose of this experimental step is to verify the experimental data and obtain more accurate results. The following specifically explains the main characteristics of each data set after the calculation is completed under the UCI system, such as the size, quantity, and name of the data. Experimental data set description is as shown in Table 1.

**Figure 1:**
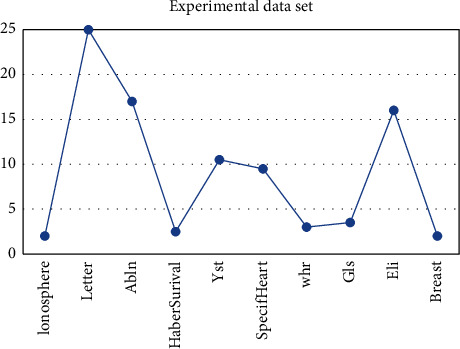
Unbalance rate of experimental data.

Through the specific analysis of the characteristics in the table, we can know that the characteristics of each data set are different, and the gap between the data sets is large, and the feedback information is also different, but the characteristics in each data set are representative. There is no duplication, and different problems in different situations are analyzed. Next, we analyzed the two columns of data specifically, namely the collection with less data and the collection with more data. We can clearly see that the collection with less data has more detailed features and is easier to process but the data are more. The features obtained from multiple sets are more general , which is more troublesome to deal with, and further analysis is needed. The unbalance rate of experimental data are as shown in Figure 1.

This article used several methods to analyze the experimental results. We used the ADA system to further verify the experimental results and then compare the latest results with the previous results to find out the rules and conclusions. In the ADA system, we did not focus on the average distribution of data but adopted more traditional calculation methods, combining experimental data with SMO calculation methods and analyzing each data set as a whole. Ignore the smaller data, and the data after the decimal point can be temporarily deleted to make the data more tidy. We can also use the RUS calculation method, which is to randomly combine the data, which will not only make the experimental data more extensive but also make the system functions better. The following table compares the centralized calculation methods. Comparison of accuracy values of the four algorithms is as shown in Table 2.

From the above table, we can know that the ADA calculation method has a higher accuracy rate and stands out in the analysis of several sets of data, and the error of the ADA calculation method is very small, and data errors or data loss are rarely seen. Therefore, in the experiment, our first recommended calculation method is the ADA calculation mode. At the same time, we have also introduced several other calculation methods to find out whether there is a better calculation mode through comparison. The specific analysis and comparison data are shown in the following table. Through a lot of analysis, we can know that the accuracy of the calculation method of RADA surpasses the ordinary ADA calculation method, becoming the best classification method for this experiment and has the highest accuracy. However, the SUI system did not combine the original data with the new conclusions in the calculation, so the accuracy of the experimental results of the system is the worst. Comparison of the recall value of the four algorithms is as shown in Table 3, Comparison of the four algorithms is as shown in Table 4.

According to the data shown in the above table, we can know that the main experimental methods are selected from the ADA calculation method, SMO calculation method, RUS calculation method, and RADA calculation method. The specific meaning value after calculation is given in the above table. Through the data, we can know that the RADA calculation method has the highest meaning value after several sets of experiments, so we can draw a more accurate conclusion: the function of the RADA system is the best, and the calculation method under this system is the simplest, and the accuracy of the experimental results is also the highest. On this basis, we analyzed the ADA calculation method, SMO calculation method, and RUS calculation method to know the ADA calculation. Although the practicability of the method is not as good as the RADA calculation method, the function is still relatively complete, and it can also be applied to the field of sports performance prediction after improvement. However, the performance of the SMO calculation method is relatively poor because the calculation method does not combine the original data with the new conclusions. Comparison of the average classification performance of the four algorithms is as shown in Table 5.

### 3.2. Establishment of a Sports Performance Prediction Model

If we want to solve some unexplainable and uncertain problems, we must use the gray forecasting model because the model can analyze smaller data based on big data, such as how to solve the number after the decimal point. In many cases, this technology has become the focus of scholars' research, and various functions are constantly being improved and gradually applied to various fields. The prediction model, as the name implies, is to predict the data. When we make data predictions, we must pay attention to not input the data into the model at one time but divide the data into different categories and then substitute them into the model, and note that the number of data in each group should not be too much; otherwise, the experimental results will be wrong. Using the gray prediction model can not only consolidate the experimental results of the above analysis but also reduce the fluctuation range of the experimental data results and determine the results to be within an easier-to-analyze range and then arrange the data according to different characteristics. The principle is as follows:

(5) $$\begin{array}{c} X^{\left(1\right)}\left(i\right)=\overset{i}{\underset{k=1}{\sum }}X^{\left(0\right)}\left(i\right)\text{.} \end{array}$$

According to the arrangement order, the formula can be obtained as follows:

(6) $$\begin{array}{c} \frac{dX^{\left(1\right)}}{dt}+aX^{\left(1\right)}=u. \end{array}$$

The specific prediction model formula is

(7) $$\begin{array}{c} {\hat{X}}^{\left(1\right)}\left(k+1\right)=\left[{\hat{X}}^{\left(0\right)}\left(0\right)-\frac{u}{\hat{a}}\right]e^{-\hat{a}k}+\frac{\hat{u}}{\hat{a}}. \end{array}$$

The GM (1, 1) model can predict nonlinear models with uncertainties in a small sample condition, which is in line with the problem of predicting college students' sports performance. However, the GM (1, 1) model has high prediction errors when there are abnormal data in the system, and it is difficult to meet the actual demand.

**Figure 2:**
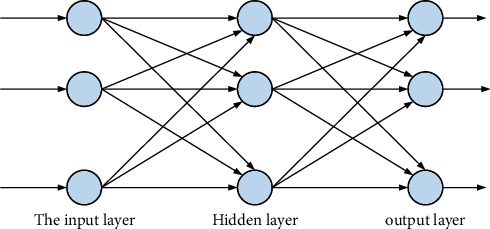
Typical BP neural network.

Neural network science refers to a science that combines specific scientific theories with the human nervous system. On this basis, we design a new model. Through the connections and interconnections between different neurons, we get specific function formula. The experimental process of this model is relatively simple, mainly including input system and output system. It can not only analyze and integrate the experimental data but also compare and predict the results of the data. Typical BP neural network is as shown in Figure 2.

The neural network algorithm system can be divided into two modes: forward transmission and reverse transmission. The specific steps are as follows: The first step is to pass the data into the system through the input system for analysis, then pass the screening layer to the more disturbing data Delete, then connect neurons with the same characteristics, then use the output system to output the conclusions of these connected neurons, and then analyze and compare the new experimental results with the initially predicted experimental results. This experimental method is forward transmission; on the contrary, if we first transfer data from the output system and then get the latest result through the input system, this method is the reverse transmission mode. The specific output formula is as follows:

(8) $$\begin{array}{c} y_{i}=\phi_{n}\left(\overset{r}{\underset{k=1}{\sum }}w_{ik}x_{k}+\theta_{i}\right). \end{array}$$

For the data output in the input system, we can express it as

(9) $$\begin{array}{c} z_{j}=\phi_{m}\left(\overset{n}{\underset{i=1}{\sum }}w_{ji}y_{i}+\theta_{j}\right). \end{array}$$

For the data output in the output system, we can express it as

(10) $$\begin{array}{c} e_{j}=\frac{1}{2}{\left(d_{j}-z_{j}\right)}^{2}. \end{array}$$

The adjusted mode for each system is

(11) $$\begin{array}{c} \delta_{ji}=\frac{\partial e_{j}}{\partial z_{j}}=\left(d_{j}-z_{j}\right)\phi \prime_{m}\left(•\right)\text{,}\\ \Delta w_{ji}=-\eta \frac{\partial e_{j}}{\partial w_{ji}}=\eta \delta_{ji}y_{i}\text{.} \end{array}$$

The specific analysis method of the filter layer is

(12) $$\begin{array}{c} \delta_{ik}=\frac{\partial e_{ji}}{\partial y_{i}}=\left(d_{ji}-y_{i}\right)\phi \prime_{n}\left(•\right)\text{,}\\ \Delta w_{ik}=-\eta \frac{\partial e_{ji}}{\partial w_{ik}}=\eta \delta_{ik}x_{k}\text{.} \end{array}$$

The advantage of the neural network system is obvious; that is, it can be well combined with other models. We can combine the neural network algorithm system with the linear calculation method to get a better performance the experimental model, but in the neural network algorithm system, there are also many problems; that is, we have to choose the input data because the system has higher requirements for data. Once the initial data are wrong, then the data brought into the model must also be wrong, which will affect the analysis of part of the data. At the same time, when using neural network algorithm systems in practice, we often encounter problems such as complicated experiment process, long experiment time, and high energy consumption. This tells us that we only use neural network algorithms. The method of the system is not feasible because the shortcomings of the system are too obvious, so in order to be able to make the experimental results more accurate, we need to combine the above-mentioned models, so as to benefit the sports performance model establishment and assessment of students' physical fitness.

The application of artificial intelligence in neural network science obviously has great advantages, which is beyond ordinary technology because artificial intelligence is more flexible in operation, requires less data conditions, and has no serious shortcomings. Therefore, this model is the best way to analyze the physical fitness of students. We used a linear model to design a new model.

In the following formula, the main variables are *W* and *B*. If we want to get a more complete experiment, we must focus on the values of *W* and *B*, so the formula we got is as follows:

(13) $$\begin{array}{c} \min\frac{1}{2}{\left\|w\right\|}^{2}+C\frac{1}{k}\overset{n}{\underset{i=1}{\sum }}\varepsilon \left(f\left(x_{i}\right)-y_{i}\right)\text{,}\\ s.t.\varepsilon \left(f\left(x_{i}\right)-y_{i}\right)=\left\{\begin{array}{c} \left|\left(f\left(x_{i}\right)-y_{i}\right)\right|-\varepsilon ,\left|w\cdot \varphi \left(x\right)+b-y_{i}\right|\geq \varepsilon \\ 0,\left|w\cdot \varphi \left(x\right)+b-y_{i}\right|<\varepsilon \end{array}\right.\text{.} \end{array}$$

In order to reduce the experimental error, we change the formula to

(14) $$\begin{array}{c} \min_{a^{\left(*\right)\propto }R^{if}}\frac{1}{2}\overset{n}{\underset{i,j=1}{\sum }}\left(a_{i}^{*}-a_{i}\right)\left(a_{j}^{*}-a_{j}\right)k\left(x_{i},x_{j}\right)+\varepsilon \overset{n}{\underset{i,j=1}{\sum }}\left(a_{i}^{*}+a_{i}\right)-\overset{n}{\underset{i,j=1}{\sum }}y_{i}\left(a_{i}^{*}-a_{i}\right)\text{.} \end{array}$$

The function of the variable can be described as

(15) $$\begin{array}{c} k\left(x_{i},x_{j}\right)=\exp\left(-\frac{{\left\|x_{i}-x_{j}\right\|}^{2}}{2\sigma^{2}}\right)\text{.} \end{array}$$

The most suitable model for the particle swarm algorithm is the pbest algorithm. We can analyze the particle performance by

(16) $$\begin{array}{c} fitness=\frac{1}{2N}{\overset{N}{\underset{i=1}{\sum }}\overset{D}{\underset{j=1}{\sum }}\left(y_{ij}-t_{ij}\right)}^{2}. \end{array}$$

The update method of the speed and position of the particles when they change is

(17) $$\begin{array}{c} v_{i\;d}^{k+1}=\varpi v_{i\;d}^{k}+c_{1}Ran\;d\left(p_{i\;d}-x_{i\;d}^{k}\right)+c_{2}Ran\;d\left(p_{ghest}^{k}-x_{i\;d}^{k}\right)\\ x_{i\;d}^{k+1}=x_{i\;d}^{k}+v_{i\;d}^{k} \end{array}$$

(1) The exercise data of students in a specific physical activity are then screened. The specific experimental steps are as follows:
  (18) $$\begin{array}{c} x_{i}^{\prime }=\frac{x_{i}-x_{\min}}{x_{\max}-x_{\min}}. \end{array}$$
(2) Input data into different models to get specific output data.
(3) The neural network system, integrated calculation method, and other modes are combined to compare the obtained results with the original data and draw conclusions.
(4) Establish a sports performance prediction model suitable for college students.
(5) Substituting the physical test scores of college students of different ages and genders into the model, and the conclusion is drawn after analysis.

**Figure 3:**
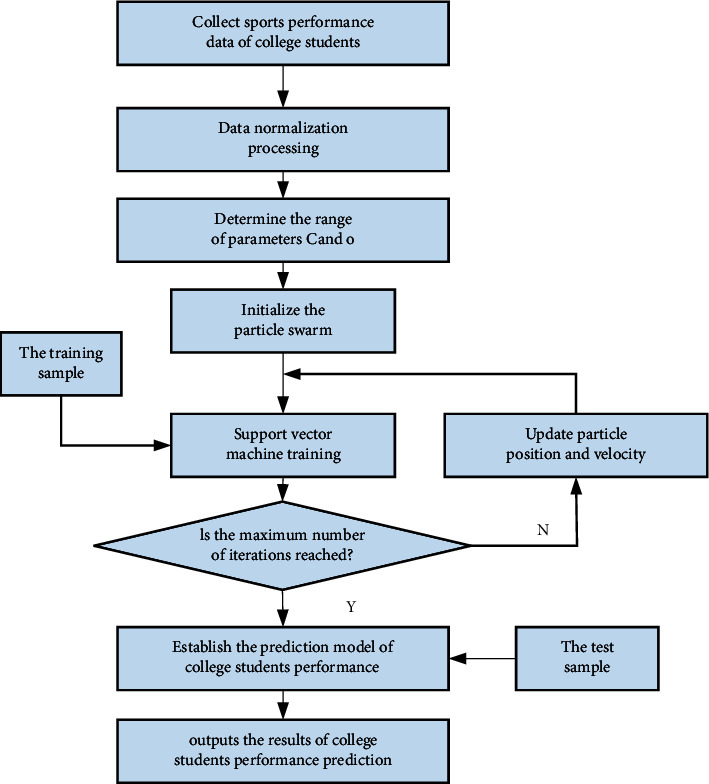
Work flow of predictive model of university students' sports performance.

The specific model operation process is as shown in Figure 3.

## 4. Results and Analysis of Sports Performance Prediction Model

### 4.1. Genetic Algorithm Prediction Model Optimization

**Figure 4:**
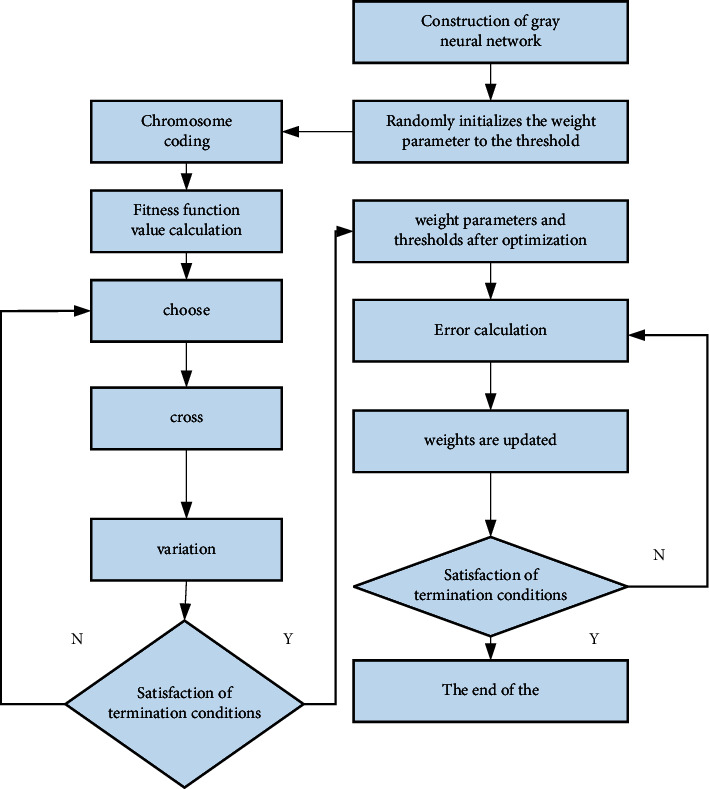
Integrated-learning algorithm model based on genetic algorithm optimization.

The advantage of the neural network system is obvious; that is, it can be well combined with other models. We can combine the neural network algorithm system with the linear calculation method to get a better performance experimental model, but in the neural network algorithm system, there are also many problems; that is, we have to choose the input data because the system has higher requirements for data. Once the initial data is wrong, then the data brought into the model must also be wrong, which will affect the analysis of part of the data and even the output of the final result. Therefore, in order to make the prediction of student sports performance more accurate, we introduced the concept of weight to make the model test faster, and the experimental results are more accurate. The integrated learning algorithm model based on genetic algorithm optimization is as shown in Figure 4.

When we substitute each data into the neural network algorithm system and integrated calculation mode, the specific function formula obtained is.


(19) $$\begin{array}{c} f_{D}=\frac{1}{\overset{n}{\underset{j=1}{\sum }}\left(d_{j}-z_{j}\right)}. \end{array}$$

### 4.2. Results and Analysis of Sports Performance Prediction

In order to prove that the newly designed predictive model is effective in analyzing the physical fitness of college students and the experimental results are correct, we concretely apply this model to a certain college and use the experimental results of the school students as a sample to test. In this test, we collected the number of sit-ups of 1,000 students. The specific data collection results are shown in the figure below. First of all, we selected 700 students as the main survey objects to obtain the exercise model of these 700 students, then tested the performance of the remaining 300 students, and then collected the data of the students with better physical fitness and poorer physical fitness data.

**Figure 5:**
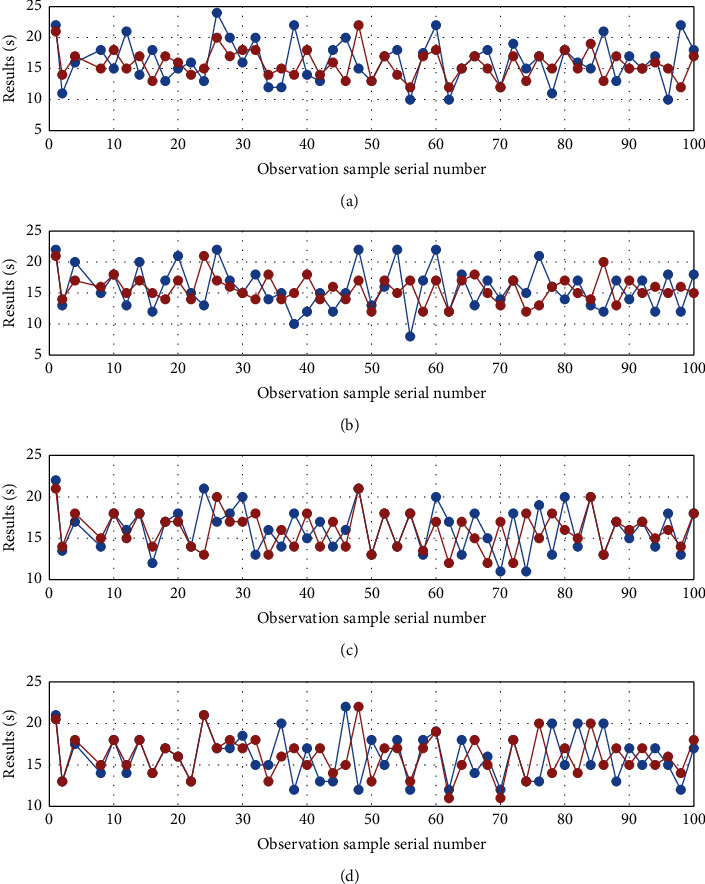
Prediction results: (a) prediction result of GM (1, 1), (b) BPNN prediction results, (c) GM-BPNN prediction results, and (d) prediction results of GA-GM-BPNN.

We selected 700 out of 1,000 experimental samples as the real experimental data, and based on the neural network algorithm system, linear calculation model, integrated calculation model, and other models, we analyzed the sports performance of the remaining 300 students. The specific experimental results are as shown in Figure 5.

According to the above data distribution, a specific conclusion can be drawn; that is, the test model we have selected can calculate and measure the physical fitness level of students if there is a big difference among them. As shown in the figure above, there are obvious signs of errors in the experimental results of the linear system model and the neural network algorithm system, but the experimental data results have more errors after comparison, so we cannot get them based on these two models and the level of physical fitness of the student. In contrast, we used the experimental method of ensemble computing model not only to predict and analyze the physical fitness level of college students but also to reduce errors and shorten the time required for the experiment. Error statistics of four prediction models are as shown in Table 6.

## 5. Conclusion

Nowadays, few people take physical exercises. In order to change this situation, we will focus on the physical activities of college students. Through the collection and analysis of college students' physical education courses, we understand that they enable students to enjoy physical activities, so how to turn these good wishes into reality has become a key research object of society and the government. Finally, the researchers decided to design a new sports performance analysis model to carry out specific experiments; in the era of rapid development of science and technology and very advanced network technology, it is not out of reach to combine the sports performance test model with artificial intelligence technology. Therefore, in order to improve the physical fitness of students, we decided to design a new model. Data collection and analysis of the students' physical education results and then the main reasons for the declining enthusiasm and physical fitness of modern college students are obtained. At the same time, this article also compared the traditional model with the newly designed model, pointed out the shortcomings of the traditional model and ideas on how to improve the method. We mainly used the integrated learning method to combine the theory with the machine to successfully establish the model and then apply this model to a university for testing. The ensemble learning model is a topic that researchers are very interested in. It has a great research and experimental value. This learning model has a huge impact on data analysis. More and more people are studying this field, and the future development prospects are good.

## Figures and Tables

**Table 1:** Experimental data set description.

Numbering	Data set	Total number of samples	Number of attributes	Less effective	Multi-like wood	Imbalance rate
1	Ionosphere	351	34	126	225	1.78
2	Letter	20000	16	789	19211	24.35
3	Abln	731	8	42	689	16.40
4	Haber survival	306	3	81	225	2.78
5	Yst	471	8	18	429	10.21
6	Specific heart	157	44	15	142	9.47
7	Wnr	186	13	48	138	2.87
8	Gls	182	9	38	144	3.79
9	Eli	336	7	20	316	15.80
10	Breast	699	9	241	458	1.90

**Table 2:** Comparison of accuracy values of the four algorithms.

Numbering	Data set	AdaBoost	SMO-boost	RUS-boost	R-AdaBoosi
1	Ionosphere	0.82	0.86	0.88	0.89
2	Letter	0.54	0.50	0.67	0.73
3	Abln	0.66	0.62	0.76	0.85
4	Haber survival	0.68	0.65	0.73	0.76
5	Yst	0.91	0.93	0.92	0.95
6	Specific heart	0.92	0.94	0.95	0.94
7	Wnr	0.74	0.7S	0.82	0.85
8	Gls	0.76	0.75	0.78	0.78
9	Eli	0.62	0.64	0.73	0.79
10	Breast	0.83	0.82	0.85	0.87

**Table 3:** Comparison of the recall value of the four algorithms.

Numbering	Data set	AdaBoost	SMO-boost	RUS-boost	R-AdaBoost
1	Ionosphere	0.81	0.80	0.81	0.87
2	Letter	0.60	0.62	0.69	0.70
3	Abln	0.65	0.64	0.69	0.72
4	Haber survival	0.62	0.61	0.65	0.69
5	Yst	0.77	0.82	0.85	0.89
6	Specific heart	0.63	0.65	0.72	0.71
7	Wnr	0.54	0.62	0.61	0.68
8	Gls	0.57	0.61	0.63	0.67
9	Eli	0.79	0–81	0.83	0.89
10	Breast	0.69	0.74	0.78	0.76

**Table 4:** Comparison of the four algorithms.

Numbering	Data set	Ada -boost	SMO-boost	RUS-boost	R-Adaboost
1	Ionosphere	0.85	0.84	0.87	0.91
2	Letter	0.66	0.64	0.75	0.74
3	Abln	0.62	0.61	0.71	0.75
4	Haber survival	0.85	0.93	0.88	0.94
5	Yst	0.94	0.96	0.93	0.96
6	Specific heart	0,75	0.79	0.83	0.87
7	Wnr	0.73	0.85	0.89	0.88
8	Gls	0.86	0.87	0.86	0.91
9	Eli	0.69	0.75	0.76	0.81
10	Breast	0.83	0.85	0.84	0.89

**Table 5:** Comparison of the average classification performance of the four algorithms.

	AdaBoost	SMO-boost	RUS-boost	R-AdaBoost
AvgPrccision	0.748	0.749	0.809	0.841
AvgRccall	0.667	0.692	0.726	0.758
AvgG-mean	0.778	0.809	0.832	0.866

**Table 6:** Table of error statistics of four prediction models.

Predictive model	RMSE	MAPE/%
GMO	0.149	11.43
BPN	0.208	14.91
GM-BPN	0.085	4.59
GA-GM-BPN	0.031	2.71

## Data Availability

The data used to support the findings of this study are available from the corresponding author upon request.
